# Genomic and Proteomic Evaluation of Tissue Quality of Porcine Wounds Treated With Negative Pressure Wound Therapy in Continuous, Noncontinuous, and Instillation Modes

**Published:** 2014-12-04

**Authors:** Kathleen L. Derrick, M. Christian Lessing

**Affiliations:** ^a^Innovation and Strategic Marketing, Kinetic Concepts, Inc, San Antonio, Tex; ^b^Scientific Affairs, KCI Medical Ltd, Dublin, Ireland, UK

**Keywords:** genomics, proteomics, negative-pressure wound therapy, hypertonic saline solution, instillation

## Abstract

**Objective:** Negative pressure wound therapy with instillation (NPWTi-d) combines NPWT with automated delivery and removal of topical wound treatment solutions. This porcine study compared genomic and proteomic responses of wounds treated with NPWTi-d with saline to wounds treated with NPWT in continuous and noncontinuous modes. **Methods:** Full-thickness porcine dorsal excisional wounds were treated with continuous NPWT, intermittent NPWT, dynamic NPWT, or NPWTi-d with saline (n = 10 wounds per group). On day 7, animals were euthanized and tissues collected. Real-time quantitative polymerase chain reaction arrays profiled expression of 84 genes including extracellular matrix remodeling factors, inflammatory cytokines and chemokines, and growth factors and major signaling molecules. Concentrations of proteins associated with angiogenesis, extracellular matrix components, and cellular energetics were analyzed via enzyme-linked immunosorbent assays. **Results:** Gene expression profiles for NPWTi-d with saline and continuous NPWT were similar. There were 5 upregulated and 18 downregulated genes overexpressed in NPWTi-d compared to NPWT wounds. Protein content was comparable in all treatment groups and similar to unwounded tissue. **Conclusions:** Previous preclinical studies have reported an increased rate of granulation tissue formation with NPWTi-d with saline compared to NPWT in continuous and noncontinuous modes. This evaluation of gene and protein expression suggests that the granulation tissue in these wounds has a similar quality. This first look at the differences in gene expression, particularly in genes related to remodeling, cell adhesion, inflammation, and growth factors, could help to clarify the observed differences in granulation rates.

Negative pressure wound therapy (NPWT) is well established for its role in reducing edema, promoting granulation, and removing exudates.^1^^,^^2^ The mechanisms of action and clinical outcomes have been evaluated in several ways, including in vitro^3^^,^^4^ and in vivo models,^5^^-^8 computer models,^9^ and randomized controlled clinical trials.^1^^,^^10^ NPWT can be delivered in continuous or noncontinuous pressure modes, and noncontinuous negative pressure can take 2 forms: intermittent NPWT (in which negative pressure alternates between a set pressure and no pressure for programmed periods of time) or dynamic/variable NPWT (with controlled transitions between high and low pressures following programmed rise and fall times).

NPWT with instillation (NPWTi-d) combines traditional NPWT with the controlled delivery and vacuum drainage of topical wound treatment solutions and suspensions, including cleansers, into and over the wound bed.^11^^,^^12^ Each NPWTi-d cycle has 3 distinct phases: instillation, dwell, and NPWT. Topical wound treatment solution or suspension is sent to the wound bed during the instillation phase. In the dwell phase, the topical wound treatment solution or suspension is held in the wound bed for a period of time. The final phase is NPWT, where solutions, suspensions, wound exudates, and infectious materials are removed from the wound bed at a user-selected interval. When this phase is complete, the cycle begins again with instillation.

In previous studies, Lessing et al^13^^,^^14^ presented the granulation response of porcine wounds treated with continuous and noncontinuous (intermittent and dynamic)^14^ NPWT, showing increased granulation rates in wounds treated with NPWTi-d with saline compared to those treated with various NPWT modes. In the first study, porcine excisional wounds treated with NPWTi-d with saline showed 43% more granulation than wounds treated with continuous NPWT after 7 days of therapy. The second study, published 2 years later, showed a 44% increase in granulation with NPWTi-d with saline compared to NPWT, as well as 57% and 40% increases compared to intermittent and dynamic NPWT, respectively. However, it was observed that, while the quantity of granulation increased, no significant differences in quality were observed histologically.

The following is a report on an exploratory analysis of the biological samples collected in the Lessing 2013 study, comparing preliminary genomic and proteomic profiles of the granulation tissue of porcine wounds treated with NPWTi-d (V.A.C. VeraFlo Therapy; KCI, San Antonio, Texas) to wounds treated with continuous and noncontinuous (intermittent and dynamic) NPWT. The purpose of this evaluation was to conduct a broad screen to identify targets for potential future studies of the mechanisms of action of NPWTi-d.

## METHODS

All animal procedures were performed under a protocol approved by the Institutional Animal Care and Use Committee at the test facility. Methods are described in the study of Lessing et al,^14^ and the study was powered to show a difference in the granulation tissue thickness, the primary endpoint of the original study. Briefly, 5 female domestic swine received 10 paraspinal (5 per side) dorsal full-thickness 5-cm diameter excisional wounds. Wound pairs were treated for 7 days with continuous NPWT (−125 mm Hg; V.A.C.Ulta Therapy System, V.A.C. GranuFoam Dressing, KCI, San Antonio, TX), intermittent NPWT (5 minutes at −125 mm Hg followed by 2 minutes at 0 mm Hg, InfoV.A.C. Therapy System, V.A.C. GranuFoam Dressing, KCI, San Antonio, Texas), dynamic NPWT (or controlled variable NPWT, with each cycle consisting of a controlled 3-minute rise to −125 mm Hg followed by a controlled 3-minute fall to −25 mm Hg; V.A.C.Ulta Therapy System, V.A.C. GranuFoam Dressing, KCI, San Antonio, Texas), or NPWTi-d (with each cycle consisting of instillation of 55 mL of sterile normal saline [instillation phase], a 5-minute soak of saline in the wound [dwell phase] and 2.5 hours of negative pressure at −125 mm Hg [NPWT phase] (V.A.C.Ulta Therapy System, V.A.C. VeraFlo Dressing, KCI, San Antonio, Texas). Cycling among these 3 phases continued for the duration of the study period. The fifth pair of wounds received an experimental negative pressure mode; however, as this mode is not clinically available, it was not included in this genomic analysis. On day 7, wound tissue biopsy samples were taken for genomic analysis to preserve RNA integrity. After euthanasia, wound tissue biopsy samples were taken for proteomic analysis.

### Genomics

Full-thickness wound tissue 6-mm biopsy samples (n = 40; 4 treatments × 2 wounds for each treatment per pig × 5 pigs) and naïve tissue (one per animal, n = 5) were collected under sterile technique, quickly removed, and stored in RNAlater (Life Technologies, Carlsbad, California) at −20°C according to the manufacturer's instructions. Total RNA was isolated from tissue using TRIzol (Life Technologies) with modifications to remove DNA using RNeasy columns and a DNase I Kit (Qiagen, Valencia, California). RNA was stored at −80°C in nuclease-free H_2_0 (Qiagen). Quality and quantity of RNA were determined using an Experion Automated Electrophoresis System (Bio-Rad, Hercules, California). First strand complementary DNA synthesis was performed using 500 ng RNA for the RT^2^ First Strand Kit (Qiagen) and stored at −20°C until ready for use. Complementary DNA was added to RT^2^ PCR Master Mix (Qiagen) and aliquoted appropriately to a 96 well Pig Wound Healing RT^2^ Profiler PCR Array (PASS-121Z; Qiagen) per manufacturer's instructions. This PCR array contains 84 key genes central to the wound healing response from each of the 3 phases of wound healing (inflammation, granulation, and tissue remodeling), including extracellular matrix (ECM) remodeling factors, inflammatory cytokines/chemokines, growth factors, and signaling molecules. Plates were run on an Applied Biosystems 7500 FAST real time PCR machine (Life Technologies) and PCR array data analysis was accomplished using Qiagen's Web-based software tool (http://pcrdataanalysis.sabiosciences.com/pcr/arrayanalysis.php).

### Proteomics

Six-millimeter biopsies were removed at time of euthanasia for protein processing, collected under sterile technique, and snap-frozen. Wound and corresponding naïve tissues biopsies were cut, weighed, and placed in Lysing Matrix D tubes (MP Biomedicals, Santa Ana, California) with 1.5-mL T-Per Tissue Protein Extraction Reagent (Thermo Fisher, Rockford, Illinois). Ten microliter of 100X Halt Protease Inhibitor Cocktail (ThermoFisher) per milliliter of lysis buffer was added immediately before use. The samples were homogenized utilizing the FastPrep 24 system (MP Biomedicals). Samples and reagents were kept on ice at all times. The samples were centrifuged and the supernatant collected for analysis. Protein concentrations were calculated by BCA (Pierce Biotechnology) and read on the BioTek (Winooski, Vermont) synergy 4 Microplate Reader at 562 nm using the Gen5 1.09 software.

Enzyme-linked immunosorbent assays were performed as per each kit's instructions for use; 1X PBS (Thermo Fisher) was used for kits without supplied diluents. Each kit was specific for the following porcine analytes from Life Sciences Advanced Technologies (St Petersburg, Florida): vascular endothelial growth factor (VEGF; n = 3), collagen type 1 (Col1; n = 3), vitronectin (VN; n = 3), fibronectin (FN; n = 5), and cytochrome C oxidase (CyCOX; n = 3). In addition, tenascin c (TNC; n = 5) was also run after preliminary genomic data results (n = 3; data not shown) were tabulated. These proteins were chosen specifically for measurement of angiogenesis (VEGF), ECM components (Col1, VN, FN, TNC), and cellular energetics (CyCOX). Samples were read on the BioTek synergy 4 Microplate Reader at 450 nm using the Gen5 1.09 software.

## STATISTICAL ANALYSIS

Values for genomics are expressed as fold regulation comparing treatment with control group. Fold changes [downregulated more than −2 fold and upregulated more than 2 fold] were defined as differentially expressed. Values for proteomics are expressed as mean ± standard error of the mean. The results were analyzed with JMP (Version 9.0.0, SAS Institute, Cary, North Carolina) and GraphPad Prism (Version 6.02, GraphPad Software, La Jolla, California). Comparisons were performed by Wilcoxon/Kruskal-Wallis. Statistical significance was defined as *P* < .05 for both genomics and proteomic analyses.

## RESULTS

Overall gene expression of the treatment groups demonstrated minor differences. For example, for NPWTi-d 58 of the 84 genes on the array (69.0%) were changed relative to naïve (*P* < .05). Both continuous NPWT and dynamic pressure control each had 56 genes (66.7%), and intermittent had 54 genes (64.3%). When looking at genes with a fold change of more than 2 or less than −2, NPWTi-d has 49 genes; 51, 52, and 47 genes round out continuous NPWT, dynamic pressure control, and intermittent, respectively (Table 1). The expression profiles of all genes on the array are listed in Table 2.

Compared to the NPWT treatment groups, the NPWTi-d group had 5 genes with a higher level of upregulation: colony-stimulating factor 2, chemokine (C-X-C motif) ligand 2 (CXCL2), interleukin 1 alpha (IL-1a), matrix metallopeptidase (MMP) 9, and TNC (Table 3). Furthermore, there were 18 more genes downregulated in NPWTi-d-treated samples as compared to the other treatment groups (fold change < −2; *P* < .05).

Cell adhesion genes such as Integrin alpha 3, Integrin beta 6 and epidermal growth factor (EGF) with its receptor EGF receptor (EGFR) were also downregulated more in NPWTi-d. Interestingly, two genes involved in the ECM were heavily downregulated in our study: E-Cadherin and MMP 7 (*P* < .05).

Interestingly, regulation trends were consistent when looking across all 4 treatment groups. For example, if a gene was upregulated for NPWTi-d, it was upregulated for the other three treatment groups. Likewise, if the gene was downregulated for NPWTi-d, then it was downregulated for all other treatment groups.

## PROTEOMICS

Key measures of angiogenesis, ECM components and cellular energetics were determined by Col1, VEGF, FN, VN, TNC, and CyCox. Although protein levels were found in each sample and treatment type, no significant difference was found between NPWTi-d and the other NPWT treated wounds (Table 4).

## DISCUSSION

NPWTi-d has traditionally been perceived as a tool for bioburden management in that wound bioburden can be diluted and removed in a cyclical basis. However, it has recently been suggested that noncontaminated and noninfected wounds could benefit from the instillation of saline. For example, Leung et al^15^ published that NPWTi-d with saline elicited a faster rate of wound fill with granulation and increased collagen content, and Lessing et al^13^ showed pronounced granulation tissue formation in porcine wounds treated with NPWTi-d with saline at a rate of 43% thicker tissue than with continuous NPWT after 7 days of therapy. However, the pivotal preclinical study by Morykwas in 1997 suggested that noncontinuous NPWT promotes a more robust granulation response than continuous NPWT.^5^ Thus, it remained unclear whether the increase in granulation tissue was due to the cleansing provided by NPWTi-d or due to the intermittent (noncontinuous) nature of NPWTi-d.

A follow-up study suggested that wounds treated with NPWTi-d with saline instillation may exhibit faster granulation rates than wounds treated with either continuous or noncontinuous NPWT.^14^ The current manuscript provides additional analysis of the tissue samples from that follow-up study.

This additional comparison of genomic and proteomic responses of NPWTi-d (using instillation foam dressing with saline) to standard NPWT with standard foam dressing, in continuous and noncontinuous modes, may help to elucidate the mechanism of the granulation rate and response of granulation, which may be tied to factors at the cellular level.

Wound healing is a complex response in which each stage consists of multiple biological processes and different cell types combining to produce relationships conducive to healing.^16^^,^^17^ Genome-wide analysis has been performed to begin the elucidation of wound-healing patterns with NPWT.^8^^,^^18^ In this porcine study, genomic analysis revealed 5 upregulated genes and 18 downregulated genes that had greater expression changes in the NPWTi-d group compared to the other NPWT groups. Discussion of roles of some of these genes is provided later.

Colony stimulating factor 2, a growth factor and cytokine, is produced by a number of cell types, including endothelial cells. Its production often requires stimulation by inflammatory agents such as IL-1. Colony stimulating factor 2 enhances antimicrobial activity of neutrophils, promotes differentiation of monocytes, and is a major stimulatory cytokine for in vitro differentiation.^19^ Since colony stimulating factors stimulate the proliferation of precursors of granulocytes and macrophages,^20^ this accentuates the wound healing continuum.

Chemokines and cytokines, like those of the CXC family, are attributed to a role for the regulation and promotion of angiogenesis as well as induction of endothelial cell proliferation in vitro.^21^ CXCL2, also known as macrophage inflammatory protein 2, has a participatory role in the early inflammation response to wounding.^22^ Because of this inflammatory response, CXCL2 is often found in association with other proinflammatory cytokines such as IL-1. Epidermal-derived proinflammatory cytokine IL-1a (IL-1a) has an interesting role in wound healing. Interleukin-1A has been produced from neutrophils, macrophages, and fibroblasts and is required to release dermal-derived inflammatory/angiogenic mediatiors.^23^ Since IL-1 has the ability to induce the expression of various growth factors and chemokines, it is assumed that they have roles in the wound healing process.^24^ However, there is a balance between inflammation and healing. In fact, Sauder et al^25^ have shown that topical IL-1a showed a statistically significant enhancement of epidermal healing.

Matrix metallopeptidase 9, a remodeling enzyme, is part of the collagenases that are known to degrade ECM components. Granulation tissue exhibits MMP9 signals by involvement in keratinocyte migration and granulation tissue remodeling.^26^ Normal wound healing requires balanced MMP activity, as MMPs also have a positive and critical role in healing and the response to injury. Matrix metallopeptidase 9 is involved in inflammation, matrix remodeling and epithelialization as well as required for normal progression of wound closure.^27^ During wound repair, expression of MMPs is upregulated possibly due to cell motility as MMP9 plays a role in cleaving basement-membrane type IV collagen, the fibrin-fibronectin clot, denatured collagen, and regeneration of the basement membrane.^28^

Finally, TNC, a cellular adhesion gene, is associated with wound healing due to its role in regulating cell proliferation, migration and differentiation within skin.^29^ Keratinocytes are reported to be a major source of TNC during the early phase of wound healing with abundant dermal TNC expression in the granulation tissue.^30^

In this study findings were also noted with two most downregulated genes unrelated to treatment and 4 NPWTi-d downregulated genes. Integrin alpha 3, Integrin beta 6 and EGF with EGF receptor (EGFR) were all downregulated more in the NPWTi-d treated samples. Integrin alpha 3, a cell surface adhesion receptor that mediates cell adhesion to the ECM, has been shown to help in the regulation of reepithelialization during wound healing.^31^ Integrin beta 6, another cell surface adhesion receptor, is an epithelial specific receptor.^32^ EGF and EGFR, as their names imply, are important in epidermal processes.^33^ This was exhibited by the current study data showing down-regulation in wound biopsies from granulation tissue compared to control biopsies from full thickness skin (including epidermis, dermis, and subcutaneous tissue). E-Cadherin and MMP7 were heavily downregulated. E-Cadherin is involved in epithelial mechanisms regulating cell-to-cell adhesion, mobility and proliferation.^34^ Matrix metallopeptidase 7 is known to play prominent roles in the injury response of mucosal epithelia^35^ and the inhibition of intestinal wound healing^36^ but interestingly is not found in cutaneous wounds.^37^ The downregulation of epithelial type molecules is not surprising given that biopsies were taken from granulation tissue.

All treatment groups trended similarly with regard to gene groupings, whether upregulated or downregulated according to the treatment regime. This trend suggests similarity between NPWTi-d and NPWT. When in NPWTi-d mode (instillation, 10-minute dwell phase, and 3.5-hour NPWT phase), the therapy system runs negative pressure approximately 95% of the time.

Six proteins were studied to better elucidate vascular and matrix quality of the resulting granulation tissue. Wound-healing-associated proteins in fibroblasts include Col1 and FN. Along with VN, they are components of the provisional matrix proteins. These proteins, as expected for normal wound healing, were found in all samples regardless of treatment. Fibronectin, important in several phases of wound healing, is crucial in ECM formation.^38^ As Prakash et al^39^ showed, no differences were seen in Col1 and FN on a healing wound even when adding additional collagen post wounding. This suggests that levels are not affected in the post-wound environment. NPWT alone has been shown to increase Col1 gene expression after just 48 hours of exposure.^40^ VEGF, known for its role as a proangiogenic factor, has been shown to play a larger role in the wound repair process. Kim et al^41^ reported that mesenchymal stem cells upregulated the early expression of MMP9, which induced early VEGF. Vitronectin has a multifunctional role in the proliferation of keratinocytes,^42^ is a major component of the ECM, and binds to several ligands including collagen and urokinase plasminogen activator receptor. Interestingly, VN was upregulated 5-fold in NPWTi-d-treated samples and was likely involved in cell migration, cell attachment, cell spreading, and hemostasis.^43^

Tenascin c, as previously stated, is linked to the wound-healing continuum. Tenascin c is seldom expressed in normal adult tissues but is consistently expressed during wound healing.^44^ Finally, CyCox is hypothesized to contribute to endothelial migration.^45^ McNulty et al^4^ found that CyCox was significantly increased following the application of NPWT, suggesting an improved energetic status.

Trends might be similar due to sampling of biopsies only on day 7 and not at earlier time points. A biopsy is a destructive sampling technique, which creates a wound within a wound that could change the original healing trajectory. Collection of biopsies at earlier time points would have required additional wounds and additional animals, which was not justifiable given the scope of the original study. As biopsy collection at intermediate time points could change the overall healing of the wound, biopsies were only collected at termination. This delay in sampling, unobtrusive to the primary outcomes of the first related study, led to conjecture regarding downstream analysis.

The genomic and proteomic observations suggest that NPWTi-d with saline may positively influence the expression of genes associated with wound healing and stimulate wound granulation with comparable vascular and matrix quality to that of continuous or noncontinuous NPWT. A recent article showed that NPWTi-d with saline has a beneficial effect in infected wounds and wounds at risk of infection.^46^ The authors proposed that, while mechanisms for increased granulation tissue formation with NPWTi-d were unclear, additional instilled fluid may reduce wound fluid viscosity, assist with the removal of exudates and infectious material, and may prompt enhanced granulation tissue formation. We hypothesize that the enhanced removal of exudates provided by NPWTi-d may contribute to the genomic and proteomic changes observed in this study.

NPWTi-d provides the mechanisms of action of NPWT along with automated delivery, dwell time, and removal of topical wound treatment solutions and suspensions. The dwell time allows for both uniform solution coverage of the wound bed^47^ and timed solution exposure to facilitate loosening of soluble debris and thinning of exudates for removal in the NPWT phase. This evaluation suggests that porcine wounds treated with NPWTi-d or NPWT in continuous and noncontinuous modes have similar expression of tissue quality-related proteins after 7 days. The observed differences in gene expression, especially in genes related to remodeling, cell adhesion, inflammation, and growth factors, may contribute to the differences in granulation rates between NPWTi-d- and NPWT-treated porcine wounds. Focused mechanistic studies that elucidate the underlying pathways of increased granulation tissue formation are warranted.

## Figures and Tables

**Table 1:** Gene expression of the 4 treatment groups: negative pressure wound therapy with instillation (NPWTi-d), continuous negative pressure wound therapy (NPWT), dynamic pressure control (DPC), and intermittent

			FC <−2, >2 and *P* < .05		
Treatment	Total no. of genes *P* < .05	Percent (%) of total genes	Total	Upregulated	Downregulated
NPWTi-d	58	69.0%	49	21	28
NPWT	56	66.7%	51	24	27
DPC	56	66.7%	52	20	32
Intermittent	54	64.3%	47	21	26

Total number of genes expressed *P* < .05; percent of the total genes expressed; Differentially expressed genes – fold change (FC) less than −2 (<−2) and greater than 2 (>2) separated into up- and downregulated genes; *P* < .05.

**Table 2:** Upregulation and downregulation (compared to naïve full-thickness skin) of all 84 genes regardless of fold change and P value

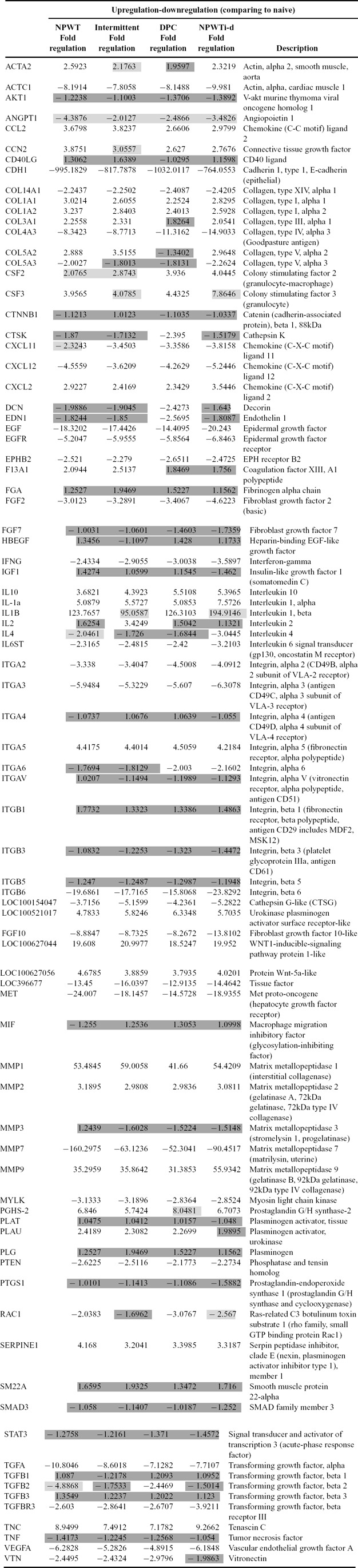

Dark boxes represent values between >−2.0 and <2.0 that are non-significant fold change. Light boxes represent non-significant (p > 0.05) values.

**Table 3:** Upregulated gene expression of negative pressure wound therapy with instillation (NPWTi-d)

Upregulated			
Gene symbol	Gene name	Fold change	Functional gene grouping
CSF2	Colony stimulating factor 2 (granulocyte macrophage)	4.04	Growth factor
CXCL2	Chemokine (C-X-C motif) ligand 2	3.54	Inflammatory cytokines and chemokines
IL-1a	Interleukin 1, alpha	7.57	Inflammatory cytokines and chemokines
MMP9	Matrix metallopeptidase 9	55.93	Remodeling enzyme
TNC	Tenascin C	9.26	Cellular adhesion

Differentially expressed genes greater than 2; *P* < .05.

**Table 4:** Protein expression of negative pressure wound therapy (NPWT), intermittent, dynamic pressure control (DPC), and negative pressure wound therapy with instillation (NPWTi-d)

Protein	NPWT	Intermittent	DPC	NPWTi-d
Col1, ng/mL per ug	20.26	18.82	20.09	21.21
VEGF, ng/mL per mg	0.72	0.97	0.58	0.63
FN, ng/mL per mg	1.38	1.38	1.16	1.16
VN, ng/mL per ug	8.99	10.62	9.75	8.57
TNC, ng/mL per mg	451.53	425.52	417.93	384.77
CyCox, ng/mL per mg	12.29	15.14	17.55	13.89

Units of protein do differ. There is no significant difference protein expression among treatment groups; *P* > .05.
